# The protein folding rate and the geometry and topology of the native state

**DOI:** 10.1038/s41598-022-09924-0

**Published:** 2022-04-16

**Authors:** Jason Wang, Eleni Panagiotou

**Affiliations:** 1 Department of Physics, University of Pennsylvania, Philadelphia, PA 19104 USA; 2 Department of Mathematics and SimCenter, University of Tennessee at Chattanooga, Chattanooga, TN 37403 USA

**Keywords:** Protein folding, Applied mathematics, Pure mathematics

## Abstract

Proteins fold in 3-dimensional conformations which are important for their function. Characterizing the global conformation of proteins rigorously and separating secondary structure effects from topological effects is a challenge. New developments in applied knot theory allow to characterize the topological characteristics of proteins (knotted or not). By analyzing a small set of two-state and multi-state proteins with no knots or slipknots, our results show that 95.4% of the analyzed proteins have non-trivial topological characteristics, as reflected by the second Vassiliev measure, and that the logarithm of the experimental protein folding rate depends on both the local geometry and the topology of the protein’s native state.

## Introduction

Proteins attain a specific conformation in space, called the native state, in order to perform their biological function. The process by which a protein attains its native state is called protein folding. Even though the mechanisms of protein folding remain largely unknown, it is possible to measure how fast a protein folds, the protein folding rate. Protein folding rates span many orders of magnitude^1^. The topomer search model suggests that proteins “search” for their native state through an ensemble of possible conformations and that folding rate is determined by the ability of the protein to reach the topology of its native state^2^. This model emphasizes the importance of the 3-dimensional structure (also known as the tertiary structure) of the native state. It is natural therefore to hypothesize that the more complex a native state is, the slower its conformation will be attained, and thus, slower the folding rate of the protein. In this manuscript, we use rigorous tools from topology to characterize the complexity of the native state (in the absence of knots), and examine the role of topology and geometry on protein folding rates.

Many measures have been used to characterize the tertiary structure of the native state and its effect in protein folding^3–22^. One of the simplest characterizations of the native state is the number of sequence distant contacts, which is the number of sequence distant amino acids which are close in 3-space^3^. This quantity has shown one of the best correlations with experimental folding rates, suggesting that it captures something relevant to protein folding. Many studies have further explored this idea, showing that, in some cases, the N- to C- termini coupling is a major determinant of the protein folding rate^23^. However, it has been difficult to create a model of protein folding based on the number of sequence distant contacts alone. The number of contacts may in fact be a proxy for a more meaningful characteristic of a 3-dimensional conformation of a protein^9^.

A rigorous framework to define conformational complexity of curves is given in knot theory, which focuses on studying simple closed curves in 3-space (knots). Topological invariants are functions defined on closed curves which can classify them in different knot types^24^. Most efforts that aim at applying rigorous notions of topology to proteins, focus on identifying knots in proteins^25–34^. Proteins, however are not closed curves; by ignoring the chemical details and simply representing a protein by its CA atoms, the native state of a protein can be seen as an open ended polygonal curve in 3-space. Previous efforts to define knotting in proteins have relied on approximating the protein by a knot (or a knotoid, which is an open knot diagram)^29,30,35–40^. This method was very successful and revealed that many proteins contain knots or slipknots (a slipknotted protein is a protein that is best approximated by an unknot as a whole, but whose subsebsegments may be best approximated by a knot^41^), but it also showed that knotted or slipknotted proteins comprise less than $2\%$ of analyzed proteins^30^. Using this method, the rest of the proteins, are all assigned a trivial topological characterization. However, the native states of the remaining proteins are not identical and their folding rates, even when comparing single domain two-state proteins, differ over many orders of magnitude. It is therefore necessary to find new ways to characterize the tertiary structure of proteins that bridge the notion of topological complexity continuously from unknotted to knotted states. A measure of complexity which does not require an artificial closure of the proteins to measure protein complexity is the Gauss linking integral. When applied over a protein or an arc of a protein, it gives the Writhe or the Average Crossing Number. It can also be used to study the linking between parts of a protein. These measures have shown strong correlation with protein folding rate and support the hypothesis that a more geometrically/topologically complex native structure leads to a lower folding rate^4,42,43^. However, the Writhe and the ACN are significantly affected by the local geometry of the protein and are not strong measures of topological complexity.

To decouple geometry from topology of proteins and capture topological effects in both knotted and unknotted proteins, with or without slipknots, we propose a new measure of the protein complexity; the second Vassiliev measure^44^. We show that the second Vassiliev measure can be applied to proteins without artificial closure of the chains to quantify topological complexity (including knotting). It takes a non-trivial value for most proteins and reflects various subtle degrees of topological complexity, varying continuously from trivial to knotted topology. In contrast to the Writhe and ACN, this tool is not affected by secondary structure elements. We apply the Writhe, the Average Crossing Number (ACN) and the second Vassiliev measure to a set of two-state proteins (which are known to fold in an all or none fashion) and a set of multi-state proteins (known to have well populated intermediates). Our results show that these measures capture different characteristics of the tertiary structure of the native state and that the folding rate depends both on the geometry and the topology of the native state, even for proteins without knots or slipknots.

## Results

In this Section, we analyze the geometry and topology of a data set of two-state and multi-state proteins whose folding rates were reported in^1,17^. More precisely, single domain protein without disulfide bonds or covalently bound ligands were considered. For the multistate kinetics, the rate constants of the rate-limiting transition were considered, if the latter was not attributed solely to cis-trans proline isomerization. In addition, the experimental temperature was in the range, or could be reliably extrapolated, to 25*C*. Each protein was represented as a polygonal curve by connecting the consecutive alpha carbon atoms, CA atoms, with a line segment. The coordinates of the CA atoms were obtained from the Protein Data Bank (PDB)^45^.

The Writhe, we denote *Wr*, and the Average Crossing Number, *ACN*, are derived from the Gauss linking integral and are very sensitive on the local geometry of proteins. The second Vassiliev measure, we denote $v_2$, and the second Absolute Vassiliev measure, we denote $Av_2$, are topological measures, quantifying the overall 3-dimensional structure, and are not as sensitive on the local geometry. Higher values of Writhe and ACN in random polygonal curves are in principle associated with higher topological complexity. In proteins however, high values of Writhe or ACN may not necessarily reflect topological complexity of the backbone, as they are significantly affected by the presence of secondary structure elements. In particular, helices contribute high values of Writhe and ACN to the total Writhe and ACN of the protein. Previous work has shown that experimental folding rates correlate with both the Writhe and the ACN of the native state^9^, but it is difficult to decouple the role of topology from the role of geometry or the role of secondary structure elements.

**Figure 1:**
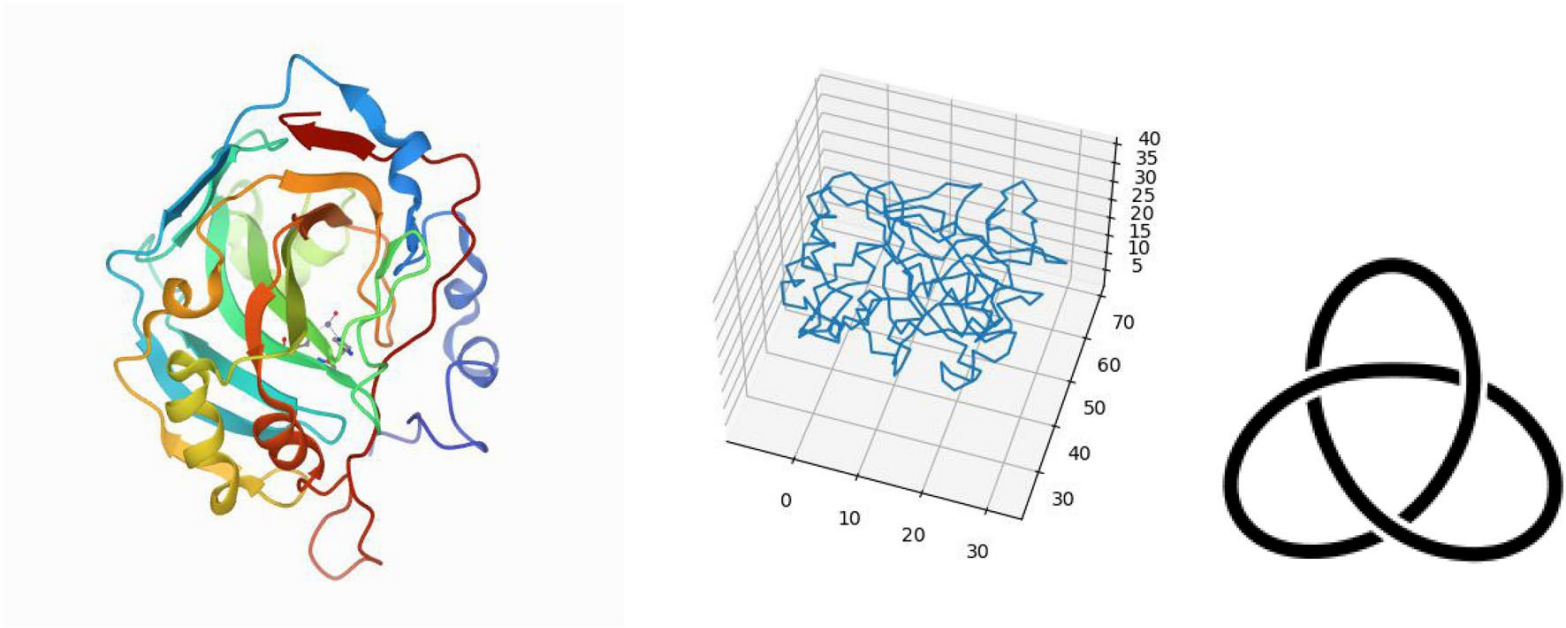
Protein 1v9e from the PDB (left) and as a simple polygonal curve (middle) and a mathematical trefoil knot (right). 1v9e has $v_2=0.808$ and $Av_2=0.844$. Note that $v_2=1$ corresponds to the trefoil knot. Indeed, 1v9e is known to contain a trefoil knot using the knot fingerprint approach^29^. Its $ACN=117.6653$ and $Wr=6.28669$.

To detect the effect of topology in protein folding, we propose using a stronger measure of topological complexity, the second Vassiliev measure, $v_2$, and the absolute second Vassiliev measure, $Av_2$. The latter is used to capture contributions to $v_2$ that cancel out because of opposite signs. $v_2$ (and $Av_2$) is not as sensitive to the local geometry as the Writhe or ACN, allowing it to capture the characteristics of the global conformation of a protein (see Fig. 1 for an illustrative example). In general, higher values of $v_2$ represent higher knotting complexity. Proteins with no knots may have values of $v_2$ that are much less than 1 in magnitude, but non-zero. Therefore, even though most proteins do not contain knots, they may be topologically not trivial, as it is reflected by non-zero values of $v_2$.

**Figure 2:**
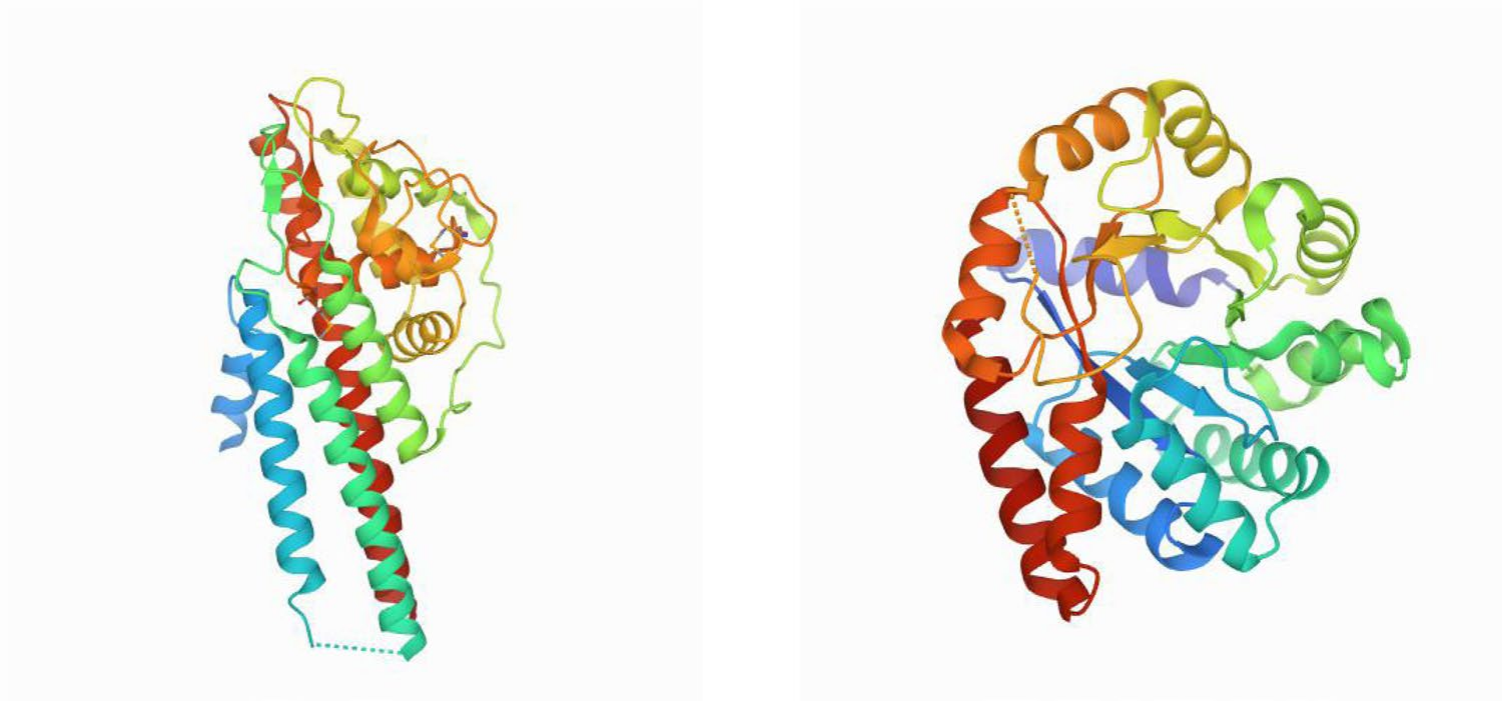
Protein 1l8w (left) and protein 1qop (right). They both have similar Writhe and ACN but different $v_2$ values. Namely, Protein 1l8w has $Wr=28.27,\ ACN=143.61$ and $v_2=0.022$. Protein 1qop has $Wr = 23.00797, \ ACN = 131.5524$ and $v_2=0.229$.

Both $v_2$ and $Av_2$ quantify how “knotted” the tertiary structure of a protein is or has the potential to be, while *Wr* and *ACN* are measures of entanglement complexity of the protein, including secondary structure element effects. These can have different values on the same protein. Examples of unknotted proteins with similar ACN and Writhe values but different $v_2$ values are shown in Fig. 2. For random chains, $Wr, ACN, v_2, Av_2$ are all expected to increase (in absolute value) with the length of a chain.

In “The topology and geometry of proteins” section we present our results on the topology and geometry of a small set of proteins. In “Folding rate as a function of the geometry and topology of the native state for a mixed (two-state and multi-state) set of proteins” section we present our results on folding kinetics and topology of the native state for a mixed set of two and multi-state proteins. In “Two-state proteins” section we focus only on the two-state proteins and in “Multi-state proteins” section we focus only on the multi-state proteins.

### The topology and geometry of proteins

We analyze a set of proteins with no knots and no slipknots. Such proteins have been “invisible” by the common mathematical methods to characterize protein conformations. However, we find that 95.4% of the proteins analyzed in this study have non-zero value for the second Vassiliev measure. Putting this result in the context of previous studies, we note that by using the knot fingerprint method and the HOMFLYPT polynomial, it has been shown that less than 2% of proteins in the PDB contain a knot^30^, while other studies show that 32% of proteins contain an “entangled motif” (two disjoint subchains with a Gauss linking integral of magnitude greater than 1)^46^. Here we provide a simple measure of topological complexity that applies to practically all proteins to characterize their topological complexity.

**Figure 3:**
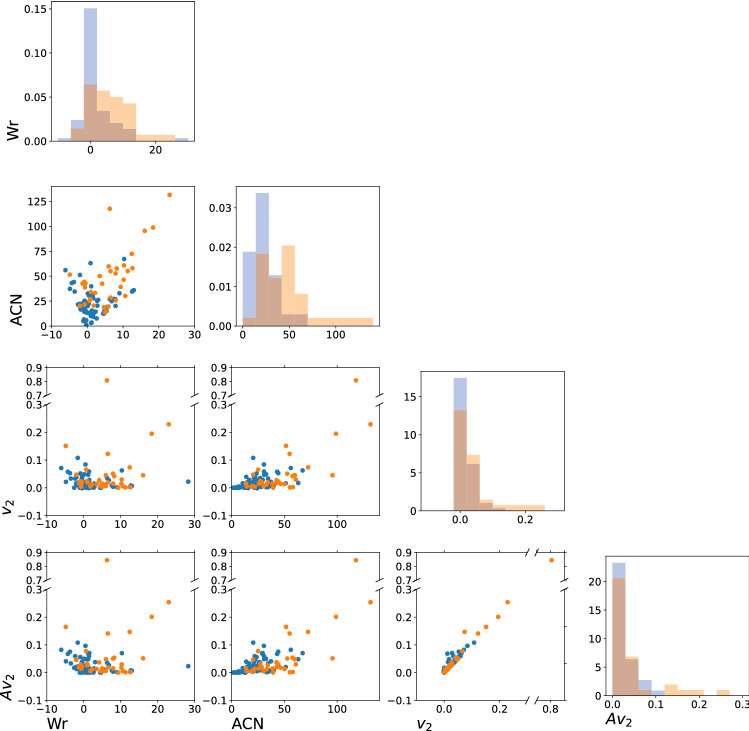
The plots on the diagonal show the distribution of each measure for the protein set. The scatter plots compare the measures pairwise for each protein. Blue points represent two-state and orange points represent multi-state proteins. We see that there is little correlation between Wr and $v_2$, Wr and $Av_2$, ACN and $v_2$, or ACN and $Av_2$. Wr and ACN show a correlation, as well as $v_2$ and $Av_2$.

We apply the second Vassiliev measure, as well as the Writhe and ACN on a set of multi-state and two-state proteins. We stress that these measures capture different conformational information. Figure 3 shows the correlation of the different topological measures used in this study. We see that the Writhe and ACN capture different information than the $v_2$ and $Av_2$. Indeed, even though the Writhe and the ACN are also measures of conformational complexity, related to topology, they are more sensitive to local entanglement rather than topology. In particular, Writhe and ACN are impacted by secondary structure elements.

### Folding rate as a function of the geometry and topology of the native state for a mixed (two-state and multi-state) set of proteins

**Figure 4:**
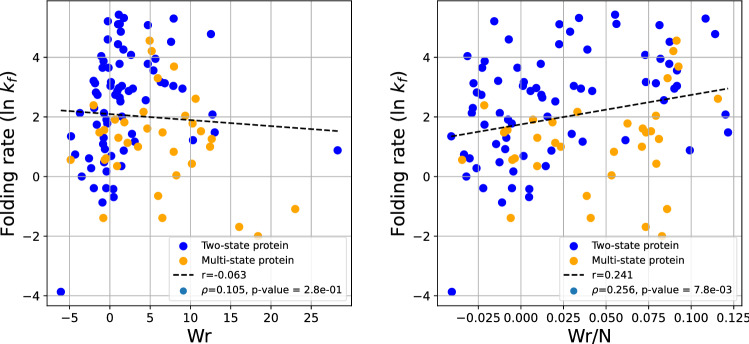
The protein folding rate as a function of the writhe (left) and writhe/N (right) for all proteins in the data set. The folding rate is represented as the natural log of the experimental folding rate $k_f$.

Figure 4 shows the logarithm of the experimental folding rate as a function of the Writhe (Left) and normalized Writhe (Right) of the native state. We see a correlation $r=0.241$ for the normalized Writhe, and the slope of the regression line is positive, which seems to contradict the hypothesis that more complex folded structures would be achieved at a slower rate. The Spearman correlation coefficient is $\rho =0.256$, with p-value $7.8\cdot 10^{-3}$, indicating a very weak correlation. Similar results were observed for a set of two-state proteins in^9^. At a closer inspection, we see that the folding rate decreases with more negative Writhe values. This was also observed in^9^. This further corroborates the result that the Writhe captures some aspects of handedness related to folding rates, as well as secondary structure elements. In particular, we know that helices contribute a positive value of Writhe. In an effort to decouple the local secondary structure effect from the topology of the protein using the Writhe, the Writhe of the primitive path was introduced in^9^ and it was shown that the folding rate correlates better with the latter. Note that previous results have also showed a different impact on folding rate of local versus global properties of the protein^47^.

**Figure 5:**
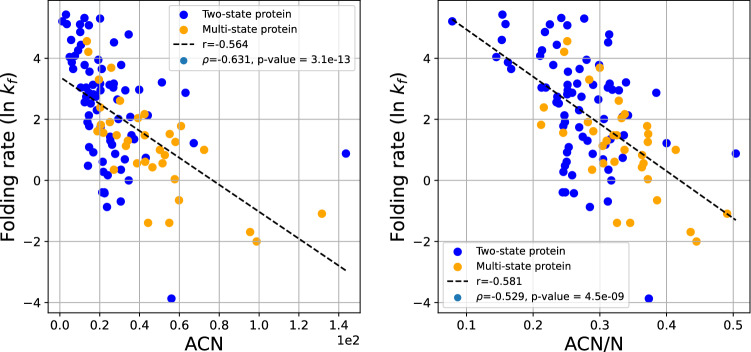
The folding rate as a function of the native state ACN (left) and the ACN/N (right) for all proteins. Multi-state proteins tend to have higher ACN than two-state proteins with similar folding rates.

Figure 5 shows the logarithm of the experimental folding rate as a function of the ACN (Left) and normalized ACN (Right). We notice that the folding rate decreases with increasing ACN and ACN/N with a correlation, $r = -0.564$ and $r = -0.581$, respectively. The Spearman correlation coefficient is $\rho =-0.631$ and $\rho =-0.529$, with p-values $3.1\cdot 10^{-13}$ and $4.5\cdot 10^{-9}$, respectively. This agrees with the hypothesis that proteins fold slower to more complex native states. However, the fact that the folding rate decreases with ACN it does not mean necessarily that it is affected by the topological complexity of the tertiary structure of the protein. The ACN, like the Writhe, is affected by the presence of secondary structures. So, the question remains to what extent the folding rate depends on the global topology versus the local entanglement of the native state.

**Figure 6:**
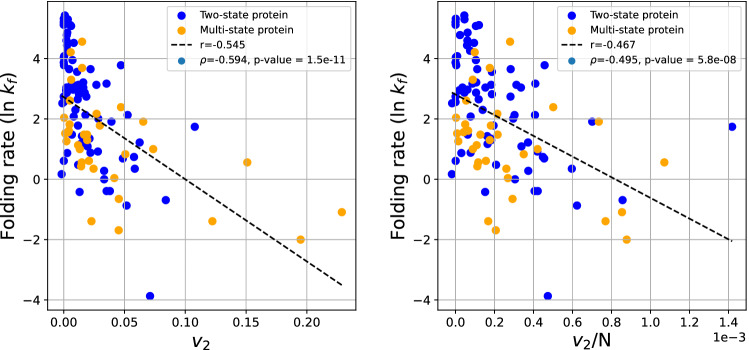
The protein folding rate as a function of $v_2$ (left) and $v_2$/N (right) for all proteins.

The second Vassiliev Measure ($v_2$) is a better indicator of topology, measuring topological complexity in the global conformation rather than being affected by local entanglement. Figure 6 shows the logarithm of the experimental folding rate as a function of $v_2$ (Right) and $v_2/N$ (Left). We see that the logarithm of the experimental folding rate decreases with increasing $v_2$ with $r=-0.545$ and $r=-0.467$, respectively. The Spearman correlation coefficient is $\rho =-0.594$ and $\rho =-0.495$, with p-values $1.5\cdot 10^{-11}$ and $5.8\cdot 10^{-8}$, respectively. This result shows that the folding rate decreases with the topological complexity of the native state. Note that this is the first result that shows that folding rate correlates with aspects of global complexity, and specifically topology, irrespective of local structure.

**Figure 7:**
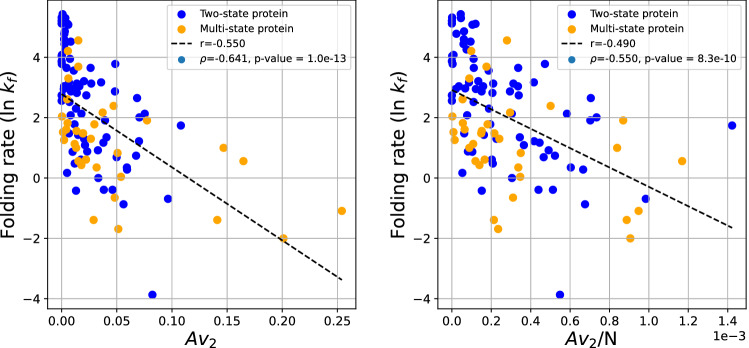
The protein folding rate as a function of the approximate $Av_2$ (left) and $Av_2$/N (right) for both two-state and multi-state proteins.

Figure 7 shows the logarithm of the experimental folding rate as a function of $Av_2$. Our results show that the folding rate has a correlation of order $r=-0.550$ and $r=-0.490$ with $Av_2$ and $Av_2/N$, respectively. The Spearman correlation coefficient is $\rho =-0.641$ and $\rho =-0.550$, with p-values $10^{-13}$ and $8.3\cdot 10^{-10}$, respectively, indicative of a moderate to strong correlation (similar to that of ACN). This is in agreement with the results on $v_2$.

### Two-state proteins

**Figure 8:**
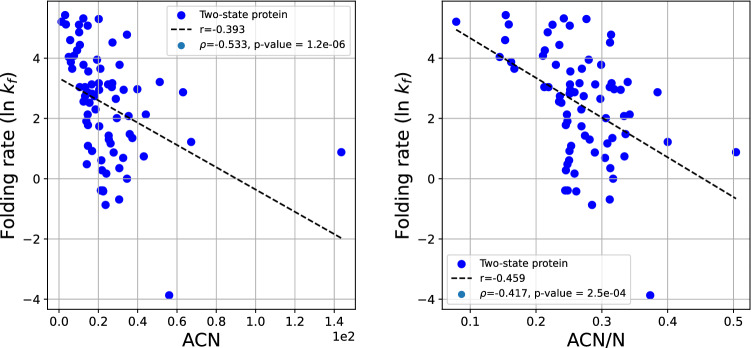
The protein folding rate as a function of the ACN (left) and ACN/N (right) of the set of two-state proteins. This set excludes the protein 1l8w which had an outlying ACN of about 140. When included the correlation decreases to r = $-0.48$.

In the set of two-state proteins, as in the combined set, the logarithm of the experimental folding rate shows a weaker than in the combined data set correlation with ACN and ACN/N, with coefficients of $r = -0.393$ and $r = -0.459$, respectively, as seen in Fig. 8. The Spearman correlation coefficient is $\rho =-0.533$ and $\rho =-0.417$, with p-values $1.2\cdot 10^{-6}$ and $2.5\cdot 10^{-4}$, respectively.

The experimental folding rate of two-state proteins and the Writhe and normalized Writhe show a Spearman correlation $\rho =0.367$ and $\rho =0.402$, with p-values $1.4\cdot 10^{-3}$ and $4.3\cdot 10^{-4}$, respectively, and a linear correlation coefficient $r=0.213$, $r=0.403$ (data shown in the SI).

**Figure 9:**
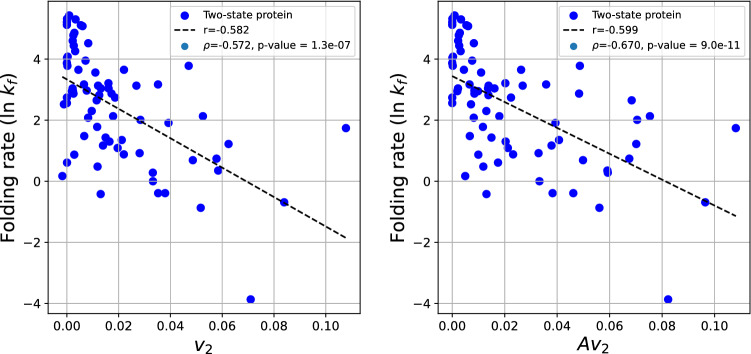
The protein folding rate as a function of the $v_2$ (left) and $Av_2$/N (right) of the set of two-state proteins.

We find that the folding rate shows a stronger correlation with $v_2$ of the two-state proteins alone, compared to the mixed set. Figure 9 shows the logarithm of the experimental folding rate as a function of $v_2$ and $Av_2$ for two-state proteins. We find that the logarithm of the experimental folding rate decreases with increasing $v_2$ and $Av_2$ values with $r=-0.582$ and $r=-0.599$, respectively. The Spearman correlation coefficient is $\rho =-0.572$ and $\rho =-0.670$, with p-values $1.3\cdot 10^{-7}$ and $9\cdot 10^{-11}$, respectively, indicative of a moderate to strong correlation, stronger than that of ACN. The normalized $v_2$ and $Av_2$ are shown in the SI.

### Multi-state proteins

**Figure 10:**
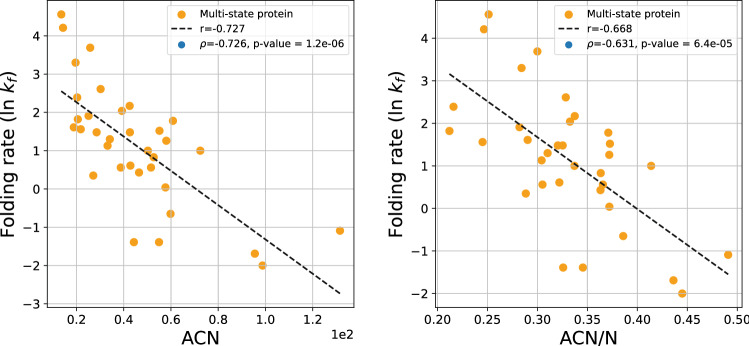
The protein folding rate as a function of the ACN and ACN/N of the set of multi-state proteins.

In the set of multi-state proteins, the logarithm of the experimental folding rate shows a particularly strong correlation with ACN, with $r = -0.727$ and $r=-0.668$, shown in Fig. 10. The Spearman correlation coefficient is $\rho =-0.726$ and $\rho =-0.631$, with p-values $1.2\cdot 10^{-6}$ and $6.4\cdot 10^{-5}$, respectively.

The logarithm of the experimental folding rate shows a weak correlation with the Writhe and normalized Writhe with Spearman correlation coefficient $\rho =-0.147$ and $\rho =0.262$, with p-values 0.41 and 0.13, respectively, and linear correlation coefficient $r=0.276$ and $r=0.214$, respectively (data shown in the SI).

**Figure 11:**
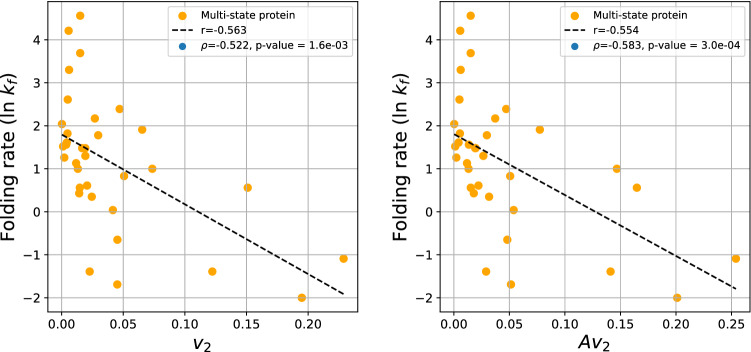
The protein folding rate as a function of the $v_2$ and $Av_2$ of the set of multi-state proteins.

Figure 11 depicts the protein folding rate against $v_2$ and $Av_2$ for multi-state proteins. The logarithm of the experimental folding rate shows a moderate correlation with these measures with $r=-0.563$ for $v_2$ and $r=-0.554$ for $Av_2$. The Spearman correlation coefficient is $\rho =-0.522$ and $\rho =-0.583$, with p-values $1.6\cdot 10^{-3}$ and $3\cdot 10^{-4}$, respectively. The normalized $v_2$ and $Av_2$ are shown in the SI.

## Discussion

Understanding protein folding requires rigorous methods for characterizing the native state of folded proteins. Many previous measures have been proposed to quantify the complexity of the native state of proteins. Even though the folding rate correlates strongly with one of the simplest characterizations of 3-dimensional complexity (the number of sequence distant contacts), the role of the topology of the 3-dimensional conformation of the entire protein in protein folding remains unclear. A reason why the role of topology in protein folding is elusive is that topological complexity has traditionally been associated with the presence of knots, but proteins with no knots or slipknots can have very different folding rates. In this manuscript, we rigorously show that folding rates depend on the mathematical topology of the native state, even for unknotted proteins. This is done using the second Vassiliev measure, a new measure of topological complexity of proteins that can characterize the topology of protein conformations continuously from trivial to knotted state. Our data show a moderate to strong correlation of the logarithm of the experimental folding rate with the second Vassiliev measure of the native state with lower folding rates associated to greater second Vassiliev measure.

We also report the ACN and Writhe values for the proteins analyzed, which are measures of conformational complexity more sensitive on the local geometry of a protein. The ACN and the Writhe are affected by the secondary structure elements, with helical proteins giving higher values. The Writhe in particular can capture some aspects of handedness as well, since right-handed turns and helices contribute a positive value, while left-handed turns contribute a negative value. Even though these characteristics may be important in understanding folding mechanisms, they can hinder our understanding of the role of the global topology of the native state in protein folding. $v_2$ and $Av_2$ on the other hand can capture the global complexity of the native state without being biased from local structure. Our results on the correlation of the logarithm of the experimental folding rate and the Writhe, ACN and the second Vassiliev measure, $v_2$ and $Av_2$, of the protein native state, show that the folding rate depends on both the geometry of the native state, as well as its topology.

The size of the proteins is a parameter already known to impact the protein folding rate^13,48–50^ and the topological complexity of a protein is expected to increase with its size. For this reason, we also report the normalized values of *ACN*, *Wr* and $v_2, Av_2$. All the Spearman correlations show a small decrease for the normalized by the length values. For the combined data set, ACN, $v_2, Av_2$ all show a similar linear correlation, while the logarithm of the experimental folding rate shows the strongest correlation with $Av_2$. In general, the folding rate of the set of 2-state and the set of multi-state proteins individually, shows a stronger linear correlation with each measure than that of the combined set, with the exception of the ACN for 2-state proteins. Such differences may be expected, as two-state and multi-state proteins have different folding mechanisms^51^. For 2-state proteins, the folding rate shows a strong correlation with $Av_2$ and a moderate to weak correlation with ACN. The folding rate of multi-state proteins, however, shows a stronger correlation with *ACN* than with $v_2$ or $Av_2$. This may suggest that, for the data set analyzed here, local structure is more involved in the folding mechanism of multi-state proteins than global topology, while the opposite is true for two-state proteins. This is in agreement with previous results on the effect of topology that were based on the contact order or the Protein Contact Network alone^52,53^. Note that the multi-state proteins attain higher $v_2$ values, indicative of more complex topology, which is expected for longer polypeptide chains. When normalized by their length however, the range of $v_2/N$ values for 2-state proteins is approximately 0 to 1.4, while for multi-state proteins is approximately 0 to 1.1. This suggests that for 2-state proteins topological complexity normalised to chain length is a more important determinant of folding rates than for multi-state proteins. On the other hand, the normalized ACN ranges from 0.1 to 0.5 for the 2-state proteins, and from 0.2 to 0.5 for the multi-state proteins. Our results thus show that for these multi-state proteins with no knots or slipknots, the rate limiting step may be associated primarily with their local geometry rather than global topology. These results may suggest that, for proteins with no knots or slipknots, the lower the topological complexity of the native state, relative to the length of a protein, the more microstates a protein can explore with the same topology and thus higher the probability to be trapped in an intermediate state.

It is possible that many of the proposed measures together could provide better correlations with protein folding rate^10^. However, in addition to correlation, it is important to establish causation. Our results show that the topology and geometry of the native state, as it is captured by rigorous and well understood mathematical tools from knot theory, should be accounted in a model of protein folding that is applicable to all proteins, with or without knots and slipknots.

## Methods

In this Section, we give the definitions of the mathematical measures used to characterize the 3-dimensional conformation of proteins.

The Writhe of a curve in 3-space is defined as the Gauss linking integral over the curve^54^:

### **Definition 1.1**

For an oriented curve $\ell$ with arc-length parametrization $\gamma (t)$, the Writhe, *Wr*, is the double integral over *l*:

1 $$\begin{aligned} Wr(l)=\frac{1}{4\pi }\int _{[0,1]^*}\int _{[0,1]^*}\frac{({{\dot{\gamma }}}(t),{\dot{\gamma }}(s),\gamma (t)-\gamma (s))}{||\gamma (t)-\gamma (s)||^3}dt ds. \end{aligned}$$

where integration is over all $s,t\in [0,1], s\ne t$.

**Figure 12:**
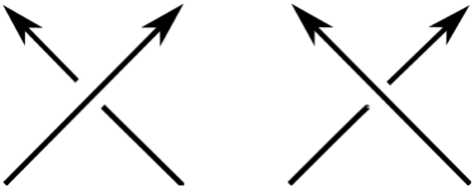
A crossing in a projection of a protein can be assigned a positive (Left) or negative (Right) sign, depending on the orientation.

The Writhe measures how much the chain turns around itself. Taking into account the orientation of the curve (from start to end-point), given a projection of the curve, one can add up the number of crossings, with signs according to orientation and the convention shown in Fig. 12. The Writhe is a real number, equal to the average algebraic sum of crossings over all possible projection directions.

By taking the absolute value of the integrand, we obtain the Average Crossing Number. Namely,

### **Definition 1.2**

For an oriented curve $\ell$ with arc-length parametrization $\gamma (t)$, the Average Crossing Number, *ACN*, is the double integral over *l*:

2 $$\begin{aligned} ACN(l)=\frac{1}{4\pi }\int _{[0,1]^*}\int _{[0,1]^*}\frac{|({\dot{\gamma }}(t),{\dot{\gamma }}(s),\gamma (t)-\gamma (s))|}{||\gamma (t)-\gamma (s)||^3}dt ds. \end{aligned}$$

where integration is over all $s,t\in [0,1], s\ne t$.

The ACN is a positive real number that measures the average sum of crossings (without signs) over all possible projection directions.

Both Writhe and ACN can be computed exactly, avoiding numerical integration, using the algorithm described in^55^. The Writhe and the ACN are continuous functions of the chain coordinates (not topological invariants) for both closed and open curves.

The second Vassiliev measure of open curves in 3-space was introduced in^44^ and is defined as follows:

### **Definition 1.3**

For an oriented curve *l* with parametrization $\gamma (t)$, $v_2$ is defined using the following integral:

3 $$\begin{aligned} \begin{aligned} v_2(l)&=\frac{1}{8\pi }\int _{0}^1\int _{0}^{j_1}\int _{0}^{j_2}\int _{0}^{j_3}(\dot{\gamma }(j_1)\times \dot{\gamma }(j_3))\cdot \frac{\gamma (j_1)-\gamma (j_3)}{|\gamma (j_1)-\gamma (j_3)|^3}(\dot{\gamma }(j_2)\times \dot{\gamma }(j_4))\\&\quad \cdot \frac{\gamma (j_2)-\gamma (j_4)}{|\gamma (j_2)-\gamma (j_4)|^3}\chi (j_1,j_2,j_3,j_4)dj_4dj_3dj_2dj_1, \end{aligned} \end{aligned}$$

where $\chi (j_1,j_2,j_3,j_4)=1$, when $(j_1,j_2,j_3,j_4)\in E$ and $\chi (j_1,j_2,j_3,j_4)=0$, otherwise and where $E\subset [0,1]^4$, such that $\Gamma (j_1,j_3)=-\Gamma (j_2,j_4)$, where $\Gamma (s,t)=\frac{\gamma (s)-\gamma (t)}{|\gamma (s)-\gamma (t)|}$, for $s,t\in [0,1]$.

The second Vassiliev measure of an open curve in 3-space is equal to the average of the algebraic sum of “alternating pairs” of crossings over all projection directions. Namely, for any given projection of the curve, an alternating pair of crossings, $(j_1,j_3)$ and $(j_2,j_4)$, is such that that if the projection of $\gamma (j_1)$ is over, resp. under, that of $\gamma (j_3)$, then the projection of $\gamma (j_2)$ is under, resp. over, that of $\gamma (j_4)$. The second Vassiliev measure does not have a closed form and can only be estimated as the average over a large number of projections. In this manuscript, $v_2$ was estimated as an average over 10,000 projections for two-state and 5,000 projections for multi-state proteins.

For closed curves, the second Vassiliev measure is a second Vassiliev invariant of knots and it is an integer topological invariant that can distinguish several knot types. For proteins, the second Vassiliev measure is a real number that is a continuous function of the chain coordinates in 3-space, and, if the protein ties a knot, it tends to the topological invariant of the knot. We note that the term “topology” in mathematics may be elusive for open curves in 3-space. It would be more accurate to use another term, such as “potential topology” for such curves. However, in this manuscript, we will use the term topological complexity, for all proteins, when $v_2\ne 0$.

Since $v_2$ is an average algebraic sum of patterns of crossings in a projection over all projection directions, positive values in one projection may cancel with negative values in another. For this reason, we introduce $Av_2$, which we define by taking the absolute value in the integrand in Eq. (3). For open curves, this is a positive number, which varies continuously with the coordinates of the chain and as the endpoints of the chain tend to coincide, it tends to the absolute second Vassiliev invariant of the resulting knot.

## Supplementary Information

Supplementary Figures.
