# Supplementary material for: The protein folding rate and the geometry and topology of the native state
Source: Sci Rep. 2022 Apr 16;12:6384. doi: 10.1038/s41598-022-09924-0 (PMC9013383; doi:10.1038/s41598-022-09924-0)
Supplement: Supplementary file 1 — Supplementary Figures. [file 41598_2022_9924_MOESM1_ESM.pdf]

The protein folding rate and the geometry and topology of the native state-Supplementary Information

Jason Wang[1] and Eleni Panagiotou*[2]

[1]Department of Physics, University of Pennsylvania, PA 19104, USA, jwang249@sas.upenn.edu
[2]Department of Mathematics and SimCenter, University of Tennessee at Chattanooga, TN 37403, USA, eleni-panagiotou@utc.edu

January 7, 2022

1 Folding rates and topological parameters

In this section we present the logarithm of the experimental folding rate as a function of the Writhe of the two-state and multi-sate proteins. We also present the normalized v_2 and Av_2 for two-state and multi-state proteins.

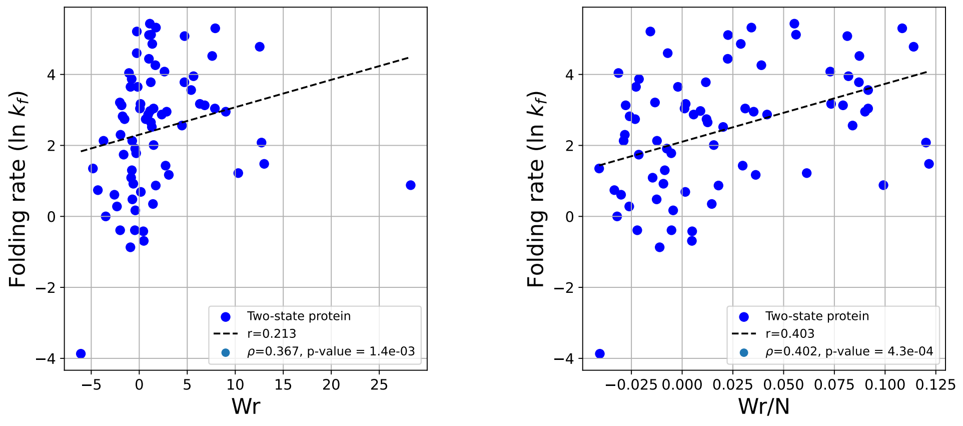

Figure S1: The protein folding rate as a function of the writhe (left) and normalized writhe (right) for two-state proteins in the data set.

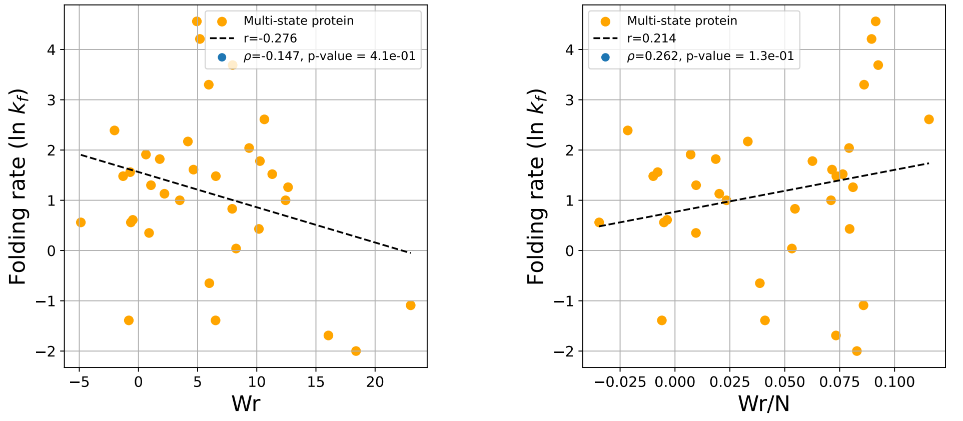

Figure S2: The protein folding rate as a function of the writhe (left) and normalized writhe (right) for multi-state proteins in the data set.

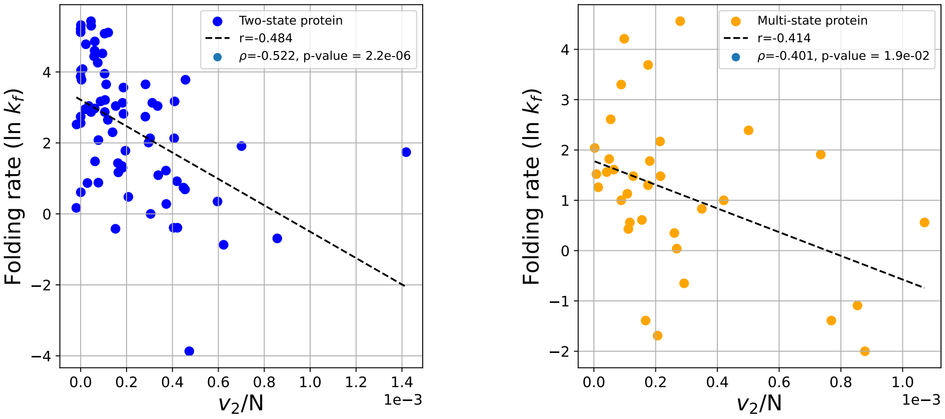

Figure S3: The protein folding rate as a function of the normalized second Vassiliev measure for 2-state (left) and mutli-state proteins (right).

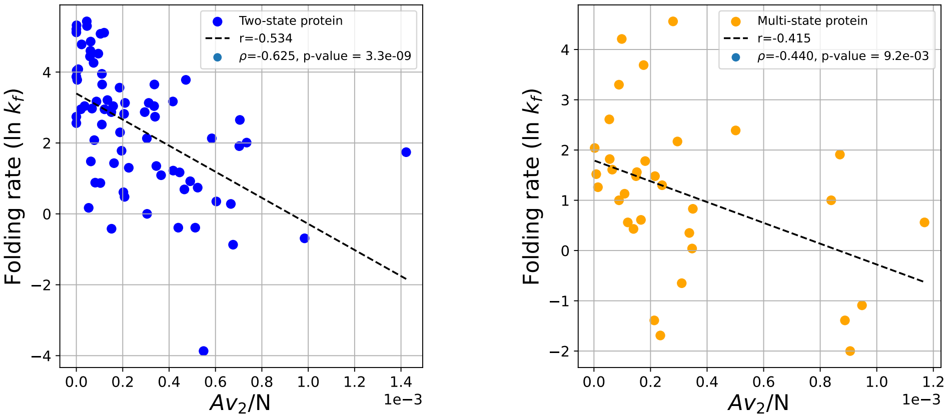

Figure S4: The protein folding rate as a function of the normalized absolute second Vassiliev measure for 2-state (left) and mutli-state proteins (right).
